# Emotional distress symptoms and their determinants: screening of non-clinical hospital staff in an Egyptian University hospital

**DOI:** 10.1186/s12888-022-04463-4

**Published:** 2022-12-15

**Authors:** Noha M. Ibrahim, Dina A. Gamal-Elden, Mohsen A. Gadallah, Sahar K. Kandil

**Affiliations:** Department of Community, Environmental, and Occupational Medicine, Faculty of Medicine, Ain Shams University, 38 Ramses St., Abbassia square, Cairo, 11566 Egypt

**Keywords:** Non-clinical hospital staff, Depression, anxiety, and stress, Work-related stress, Socioeconomic status, Effort-reward imbalance, Income insufficiency, Economic conditions and mental health, mental health

## Abstract

**Background:**

Non-clinical hospital staff were rarely studied despite their potential exposure to workplace stressors. We aimed to measure the prevalence of depression, anxiety, and stress (emotional distress symptoms) and determine their association with perceived job stress level and socioeconomic factors among non-clinical hospital staff.

**Methods:**

This cross-sectional study was conducted in Ain-Shams University Hospitals from March to May 2019. Tools were the Arabic Depression, Anxiety, and Stress Scale-21, Workplace Stress Scale, and Socioeconomic status scale. Independent correlates were determined using multivariable ordinal regression.

**Results:**

Out of 462 participants, 72.5% reported receiving insufficient income and 54.8% showed Effort-reward imbalance. Job stress was scored as severe/potentially dangerous by 30.1%. The prevalence of depression, anxiety, and stress were 67.5, 69.0, and 51.7%; and the severe/extremely severe levels were 20.8, 34.6, and 17.6% respectively. Across all the severity levels, the likelihood of depression, anxiety, and stress were progressively higher with more serious levels of income insufficiency [in debt versus able to save, OR:5.82 (95%CI:2.35–14.43), OR:3.84 (95%CI:1.66–8.91), and OR:3.01 (95%CI:1.20–7.55) respectively] and with higher job stress levels. Specifically, the likelihood of depression, anxiety, and stress increased by 74, 56, and 53% respectively with feelings of unpleasant/unsafe work conditions and by 64, 38, and 62% respectively with the presence of work-life conflict; while the likelihood of depression and stress increased by 32 and 33% respectively when there was difficult communication with superiors; and only the likelihood of depression increased by 23% with underutilization of skills.

**Conclusion:**

Non-clinical hospital staff were commonly affected by emotional distress symptoms with high rates of severe/very severe levels, and they often considered their workplace stress as severe/potentially dangerous. Workplace stress and income insufficiency were strong correlates with emotional distress symptoms. Decreasing work-life conflict, enhancing leadership skills, and mitigation of the economic hardship are needed.

**Supplementary Information:**

The online version contains supplementary material available at 10.1186/s12888-022-04463-4.

## Introduction

Mood disorders cause a large disease burden and loss of productivity in the population. According to the Health and Safety Executive national annual statistics 2019/2020 in Great Britain, 51% of cases of work-related ill health and 55% of working days lost were attributed to depression, anxiety, or stress [1]. In Egypt, the estimated national prevalence of mood and anxiety disorders were 6.4 and 4.8% respectively and the overall mental disorders were 11.5% among professionals [2]. In contrast, symptoms of depression, anxiety, and stress (DAS) were much more reported in the population and the severity levels of each represent a continuum whose extreme signifies the genuine clinical disorder [3]. Early detection of such symptoms could enable taking proactive preventive and/or curative measures particularly in critically demanding workplaces like hospitals. The healthcare industry is often known by its work-related stress factors. The rapid work pace and low threshold of tolerance for mistakes can create an atmosphere of continuous stress. Hospital workplace was rated as very/extremely stressful by 26% of hospital staff in an Iranian study including both clinical and non-clinical [4], and as high as 46.7% of oncologists scored their job stress level as severe/potentially dangerous in an Egyptian study [5]. Compared to other workers, healthcare professionals have a greater chance of suffering symptoms of depression, anxiety, stress, sleep disturbance, or even the genuine clinical psychiatric disorders [1, 6]. On the other hand, financial hardship or threat and the feeling of effort-reward imbalance were also strongly associated with such symptoms [7–11]. Contextually, Egyptian economy has been greatly impacted particularly in years 2017 to 2019 due to the currency devaluation with the resulted high rate of inflation [12]. In workplaces like hospitals, such an economic situation may cause a state of imbalance between a high level of education and job demand on the one hand and the sufficiency of income taken in return on the other hand.

The reported proportions of DAS symptoms among clinical hospital staff were approximating 35% for depression and 40% for anxiety and stress [13, 14] and symptoms of depression were reported by 7.5% of non-clinical hospital staff in a Nigerian study [15]. Additionally, the pooled prevalence conveyed in a meta-analysis among hospital staff other than physicians and nurses were 20.6, 27.0, and 36.4% for DAS respectively while among physicians and nurses, were 24.3, 25.8, and 45% respectively [16]. Among employed individuals in a population-based Iranian study, DAS symptoms were reported by 23.7, 26.3 and 30.3% respectively [17] and by 19.7, 33.1, and 10.6% respectively among government employees in an Indian study [18]. In Egyptian research however, the job categories included for studying emotional distress symptoms were schoolteachers [19] and medical students in studies published before the date of our study conduct in year 2019 [20, 21]; and the clinical hospital staff in studies done in subsequent years [22, 23]. Seemingly, non-clinical hospital staff (NCHS) were rarely studied despite their potential vulnerability to hospital work stress; besides, they are usually not sufficiently rewarded—both financially and emotionally—compared to their clinical counterpart and hence, we targeted this group of overlocked hospital workers. This study aimed to measure the prevalence of DAS and to determine their association with socioeconomic factors and perceived job stress level among NCHS. Research questions were: what is the perceived job stress level among NCHS? What is the prevalence of suffering DAS symptoms in their various severity levels and what are their independent risk factors in-terms of socioeconomic status, income, perceived job stress level, and specific work stressors?

## Methods

### Study design, setting, and sample

A cross-sectional study was conducted among NCHS of Ain-Shams University Hospitals (ASUHs) from March to May 2019. ASUHs, one of the largest Egyptian university hospitals, consists of a medical campus including 12 hospitals and specialized centers. The study population was the total workforce of NCHS who have been working for at least 1 year (*N* = 3502). Non-clinical jobs were categorized into 4 groups: the administrative [clerks, accountants, secretary, admission office/medical record department staff, and public affairs (*N* = 2515)], technicians [chemists, laboratory, radiology, and maintenance technicians (*N* = 592)], engineers/information technology (IT) professionals (*N* = 146), and unskilled workers (*N* = 249). A sample of 462 participants was calculated as sufficient to measure the prevalence of DAS among staff based on 40% estimated prevalence of emotional distress [14] with a 5% precision, 95% confidence level, and accounting for 20% non-response rate. All NCHS who have been working for ≥1 year were invited to participate with no other inclusion or exclusion criteria. Study tools were distributed in all hospitals as a paper form self-administered questionnaire to on-the-job NCHS. The number of participants recruited from each job category was determined proportional to its total workforce number.

### Study tools

A structured standardized questionnaire was used after being pilot tested on a sample of NCHS (*n* = 20) and no changes were made. The questionnaire consisted of the following sections:

#### Section I: measuring socio-economic status

The structured standardized and validated socioeconomic status scale for health research in Egypt was used. This tool has 7 domains first, education and cultural domain (scores ranged from 0 = illiterate to 14 = post graduate degree through 8 educational levels, in addition to 1 point for adequate access to health information for each of husband and wife. The total domain score = 30. Second, occupation domain (none-working/housewife = 0, to professionals = 5 through 6 levels for each of husband and wife) The total domain score = 10. Third, family domain including residence (slum = 0, rural = 1, urban = 2), number of family members (≥5 = 1, < 5 = 2), number of earning family members (1 = 1, 2 = 2, ≥3 = 3), education of children aged ≥5 years (all = 3, ≥50% = 2, < 50% = 1, none of them = 0). The total domain score = 10. Fourth, family possessions domain (having some items and owning of real estates, lands, farms, …etc.). The total domain score = 12. Fifth, economic domain (in debt = 0, just meeting routine expenses = 1, meeting routine expenses and emergencies = 2, able to save/invest money = 3, not receive governmental support = 1, pays tax = 1) The total domain score = 5. Sixth, home sanitation domain including services (e.g., water, electricity, natural gas, … etc. =7), type of house (owned ≥4 rooms = 4, owned < 4 rooms = 3, rented ≥4 rooms = 3, rented < 4 rooms = 1, no place to live = 0), crowding index (≤1 person/room = 1, >1person/room = 0) The total domain score = 12. Seventh, health care domains (private facilities = 5, health insurance = 4, free governmental health service = 3, more than one of the above sources = 2, traditional healer/self-care = 1) The total domain score = 5. The total scale score = 84. In cases with non-applicable items, the total score was multiplied by a correction factor. Total score was divided into four levels by quartiles as follows: very low (≤21), low (22–42), middle (43–63) and high (≥64) [24].

#### Section IΙ: measuring depression, anxiety, and stress (DAS)

The standardized Arabic translation of the Depression Anxiety Stress Scale (DASS-21), the short version of DASS-42, was used [25]. It has 21 items in three sub-scales, 7 items for each. The scale quantitatively measures the severity of, and distinguishes between, emotional states of depression, anxiety, and stress with very high internal consistency. Reliability α coefficients for the three subscales were 0.93, 0.90, and 0.93 for depression, anxiety, and stress respectively [3]. Its design assumed that emotional symptoms vary along a continuum of severity in which genuine clinical disorders represent the extreme and pathological manifestation of basic emotional states encountered in non-clinical general population [3]. Depression sub-scale assesses dysphoria, hopelessness, devaluation of life, self-deprecation, lack of interest/involvement, and anhedonia/inertia. Anxiety sub-scale assesses autonomic arousal, skeletal muscle effects, situational anxiety, and subjective experience of anxious or panic affects. Stress sub-scale measures chronic non-specific arousal. It assesses difficulty relaxing, nervous arousal, and being easily upset/agitated, irritable/over-reactive and impatient. Participants were inquired about experiencing any of the symptoms within the last week.

The rating scale for each item included 0 = Did not apply to me at all, 1 = Applied to me to some degree/or some of the time, 2 = Applied to me to a considerable degree/or a good part of time, 3 = Applied to me very much/or most of the time. Scores for subscales were calculated by summing the scores for the relevant items and multiplied by two to get scores equivalent to DASS-42. Severity levels were categorized as normal, mild, moderate, severe, and extremely severe according to the scoring manual. For depression, anxiety, and stress respectively, the score ranges were 0–9, 0–7, and 0–14 for Normal; 10–13, 8–9, and 15–18 for Mild; 14–20, 10–14, and 19–25 for Moderate; 21–27, 15–19, and 26–33 for Severe; and ≥ 28, ≥20 and ≥ 34 for Extremely severe levels [26]. Participants who suffered severe and extremely severe levels were directed to seek psychiatric consultation and for those who agree, arrangement for such consultation was provided with the help of the study team.

#### Section ΙII: assessment of perceived job stress level

Workplace Stress Scale (WSS) [27] was used to measure perceived job stress regarding psychosocial work environment. It was designed as a quick eight-items test describing how often participants experience certain feelings in their current job. Items were: 1) feelings of unpleasant/unsafe work conditions, 2) feeling that job negatively affect physical/emotional wellbeing, 3) having much work and/or unreasonable deadlines, 4) having difficulty to express opinions/feelings about job conditions to superiors, 5) feeling that job pressures interfere with family/personal life, 6) having adequate control/input over work duties, 7) receiving appropriate recognition/rewards for good performance, and 8) the ability to utilize skills/talents to full extent at work. Responses were rated on a five-point Likert scale. Scoring for items from 1 to 5 was “never” = 1 to “very often” = 5 and reversed for items from 6 to 8 (“never” = 5 to “very often” = 1). The sum of scores categorized job stress level into relatively calm (≤15), fairly low (16–20), moderate (21–25), severe (26–30), and potentially dangerous (31–40).

### Definition of variables

*“Receiving insufficient income”* was considered with responses of “in debt” and “just meeting routine expenses” for the question inquiring about income. *“Having much workload”* and *“Not receiving appropriate recognition/rewards”* were considered with responses “sometimes”, “often” or “very often” for item-3 and “never” or “rarely” responses for item-7 of WSS respectively. Presence of *“Effort-reward imbalance”* was considered when participants were having much workload and either receiving insufficient income (*Financial Effort-reward imbalance*), not receiving appropriate recognition/rewards (*Emotional Effort-reward imbalance*), or both. *“Emotional distress”* was an operational term used in this study to collectively indicate depression, anxiety, and stress.

### Statistical analysis

Description was done using frequencies and proportions for categorical variables and median, interquartile range (IQR), minimum and maximum for scale variables. For the preliminary bivariate analysis, the outcome variables were dichotomized as normal versus other severity levels of DAS. Binary logistic regression was used to calculate the unadjusted odds ratios (OR), their 95% confidence intervals (95%CI), and the *P*-values. To determine the independent associations between exposures and outcome variables, multivariable ordinal regression models were constructed for each of DAS as ordinal outcomes. This type of modeling was chosen to enable estimating the odds of being at or above a given severity level across all cumulative splits of the outcome variables. It has the advantage of being parsimonious because it makes only a single model for each variable instead of making multiple separate logistic regression models analogous to the sequence of splits within the various severity levels [28]. Hence, the calculated effect size for each independent variable denoted its effect across all the severity levels of the dependent variables. Ordinal regression requires the proportional odds assumption to be met. Models included variables that showed significant association on bivariate analysis (*P*-value ≤0.05). Two models were constructed for each outcome: Model-1 included age, gender, job category, income, and perceived job stress level. Model-2 aimed to clarify the job stress items that specifically associated with the outcome. It included age, gender, job category, income, and the eight items of WSS. Age and WSS items were treated as continuous covariates. Proportional odds assumption was tested for each model. Correlates of severe/potentially dangerous level of job stress and of insufficient income were also determined using binary and multivariable logistic regression analysis; to explore differences in the presence of such exposure variables particularly within job categories. Adjusted OR and the effect estimates were provided with their 95%CI and the exact *P*-values. *P*-value ≤0.05 was considered significant. Reliability analyses were performed, and Cronbach’s alpha values were 0.817, 0.814, 0.793, and 0.721 for depression, anxiety, stress, and WSS items. All analyses were done using SPSS version 25.

### Ethics statement

This study was approved by Ain Shams University Faculty of Medicine Research Ethics Committee (approval number FMASUP15b/2019). This study was performed in accordance with the ethical standards of the Declaration of Helsinki, 1964 and its later amendments. Informed consents were obtained from all participants. Confidentiality was kept and no personally identifying data were presented.

## Results

### Demographics and economic status

This study included 462 NCHS, their median age was 45 years (IQR: 34–52), and women were 63.0% (291/462). Most of them achieved university or higher education (60.4%, 279/462), belonged to the middle (79.2%, 366/462) and high social class (16.9%, 78/462), and worked in administrative job (74.5%, 344/462). Only unskilled workers were underrepresented as many of them refused to participate (their response rate = 51.5% (17/33) of the calculated required number) while refusal in other job categories was null. Almost three quarters (72.5%, 335/462) reported receiving insufficient income [in debt = 21.0% (97/462) and just meeting routine expenses = 51.5% (238/462)] (Table 1), and this proportion was significantly lower among staff with university/higher education than the less educated [68.5% versus 78.7%, (OR:0.52, 95%CI:0.32–0.83)] and significantly higher among administrative staff than engineers/IT professionals [74.7% versus 50.0%, (OR:2.89, 95%CI:1.06–7.87)] (Supplementary Table 1, Additional file 1).

**Table 1:** Distribution of the presence of emotional distress symptoms by demographics, job stress level, and Effort-reward imbalance (*N* = 462)

	Total	Depression (any level) *n*/total (row %)	Anxiety (any level) *n*/total (row %)	Stress (any level) *n*/total (row %)
	***N***. = 462	312/462 (67.5)	319/42 (69.0)	239/462 (51.7)
	*n*. (column %)	95%CI (63.2–71.7%)	95%CI (64.7–73.1%)	95%CI (47.1–56.3%)
**Age:** Median = 45 (IQR:34-52, Min. = 18-Max. = 60)				
18 – 30 years	70 (15.2)	51/70 (72.9)	52/70 (74.3)	46/70 (65.7)
31 – 45 years	164 (35.5)	115/164 (70.1)	114/164 (69.5)	95/164 (57.9)
46 – 60 years	228 (49.4)	146/228 (64.0)	153/228 (67.1)	98/228 (43.0)
**Gender**				
Men	171 (37.0)	110/171 (64.3)	104/171 (60.8)	91/171 (53.2)
Women	291 (63.0)	202/291 (69.4)	215/291 (73.9)	148/291 (50.9)
**Marital status**				
Un-married	89 (19.3)	62/89 (69.7)	58/89 (65.2)	46/89 (51.7)
Married	373 (80.7)	250/373 (67.0)	261/373 (70.0)	193/373 (51.7)
**Residence**				
Rural/urban slum	60 (13.0)	36/60 (60.0)	46/60 (76.7)	38/60 (36.3)
Urban	402 (87.0)	276/402 (68.7)	273/402 (67.9)	201/402 (50.0)
**Educational level**				
Less than university	183 (39.6)	125/183 (68.3)	128/183 (69.9)	95/183 (51.9)
University or higher	279 (60.4)	187/279 (67.0)	191/279 (68.5)	144/279 (51.6)
**Job categories**				
Workers	17 (3.7)	12/17 (70.6)	12/17 (70.6)	12/17 (70.6)
Technicians	81 (17.5)	60/81 (74.1)	59/81 (72.8)	49/81 (60.5)
Administrative	344 (74.5)	232/344 (67.4)	237/344 (68.9)	171/344 (49.7)
Engineers/IT professionals	20 (4.3)	8/20 (40.0)	11/20 (55.0)	7/20 (35.0)
**Income**				
In debt	97 (21.0)	82/97 (84.5)	78/97 (80.4)	65/97 (67.0)
Just meet routine expenses	238 (51.5)	167/238 (70.2)	169/238 (71.0)	125/238 (52.5)
Meet routine and emergency expenses	100 (21.6)	52/100 (52.0)	60/100 (60.0)	39/100 (39.0)
Able to save/invest money	27 (5.8)	11/27 (40.7)	12/27 (44.4)	10/27 (37.0)
**Socioeconomic status**				
Low	18 (3.9)	12/18 (66.7)	14/18 (77.8)	14/18 (77.8)
Middle	366 (79.2)	249/366 (68.0)	257/366 (70.2)	190/366 (51.9)
High	78 (16.9)	51/78 (65.4)	48/78 (61.5)	35/78 (44.9)
**Job Stress Level**				
Relatively Calm	57 (12.3)	19/57 (33.3)	25/57 (43.9)	12/57 (21.1)
Fairly Low	117 (25.3)	60/117 (51.3)	67/117 (57.3)	31/117 (26.5)
Moderate	149 (32.3)	105/149 (70.5)	107/149 (71.8)	84/149 (56.4)
Severe	104 (22.5)	95/104 (91.3)	88/104 (84.6)	82/104 (78.8)
Potentially Dangerous	35 (7.6)	33/35 (94.3)	32/35 (91.4)	30/35 (85.7)
**Effort-reward imbalance**				
No	209 (45.2)	116/209 (55.5)	123/209 (58.9)	78/209 (37.3)
Yes (any)	253 (54.8)	196/253 (77.5)	196/253 (77.5)	161/253 (63.6)
Financial	229 (49.6)	179/229 (78.2)	179/229 (78.2)	145/229 (63.3)
Emotional	146 (31.6)	114/146 (78.1)	118/146 (80.8)	102/146 (69.9)
Both	122 (26.4)	97/122 (79.5)	101/122 (82.8)	86/122 (70.5)

*IQR* interquartile range, *Min* minimum, *Max* maximum, *IT* Information Technology

### Job stress level

**Fig. 1:**
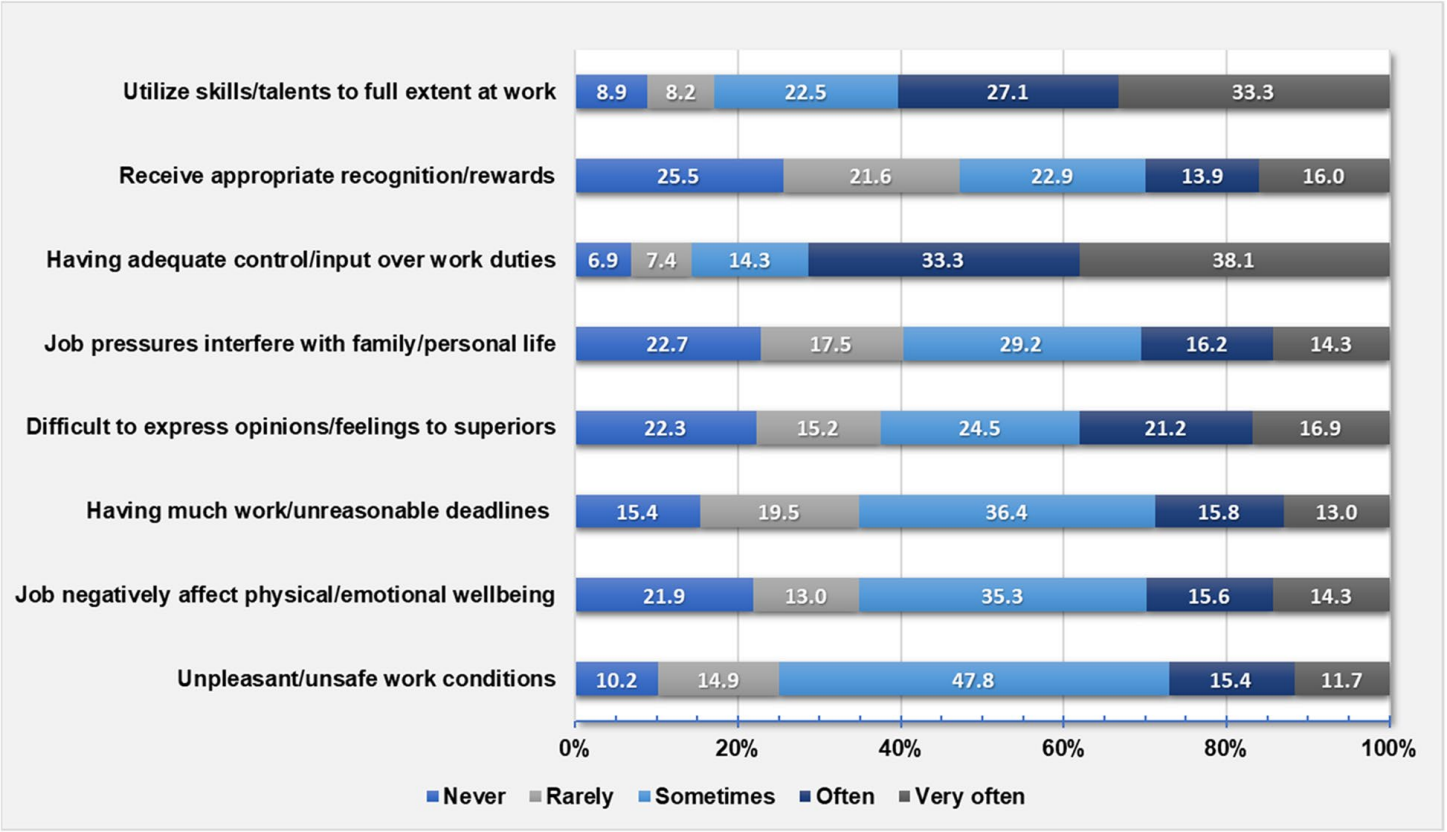
Proportions of experiencing Workplace Stress Scale items (*N* = 462)

A total of 30.1% (139/462) of NCHS scored their job stress as severe (22.5%, 104/462) and potentially dangerous (7.6%, 35/462) (Table 1). The prevalence of severe/potentially dangerous job stress showed no significant differences between job categories (Supplementary Table 2, Additional file 1). Effort-reward imbalance was found in 54.8% (253/462) of NCHS [financial in 49.6% (229/462) and emotional in 31.6% (146/462)] (Table 1). Frequencies of experiencing individual job stress items were depicted in (Fig. 1). In a frequency of sometimes/often/very often, three quarters of participants considered their work condition as unpleasant/unsafe and almost two thirds felt that job negatively affected their physical/emotional wellbeing, had much workload and/or unreasonable deadlines, felt their job interfered with their lives, and found difficulty in communicating opinions/feelings to superiors. On the other hand, control over work duties and the full utilization of skills were sometimes/often/very often felt by 85.7 and 82.9% respectively, while appropriate recognition or rewards was sometimes/often/very often received in 52.8% of staff.

### Emotional distress

#### Prevalence

**Fig. 2:**
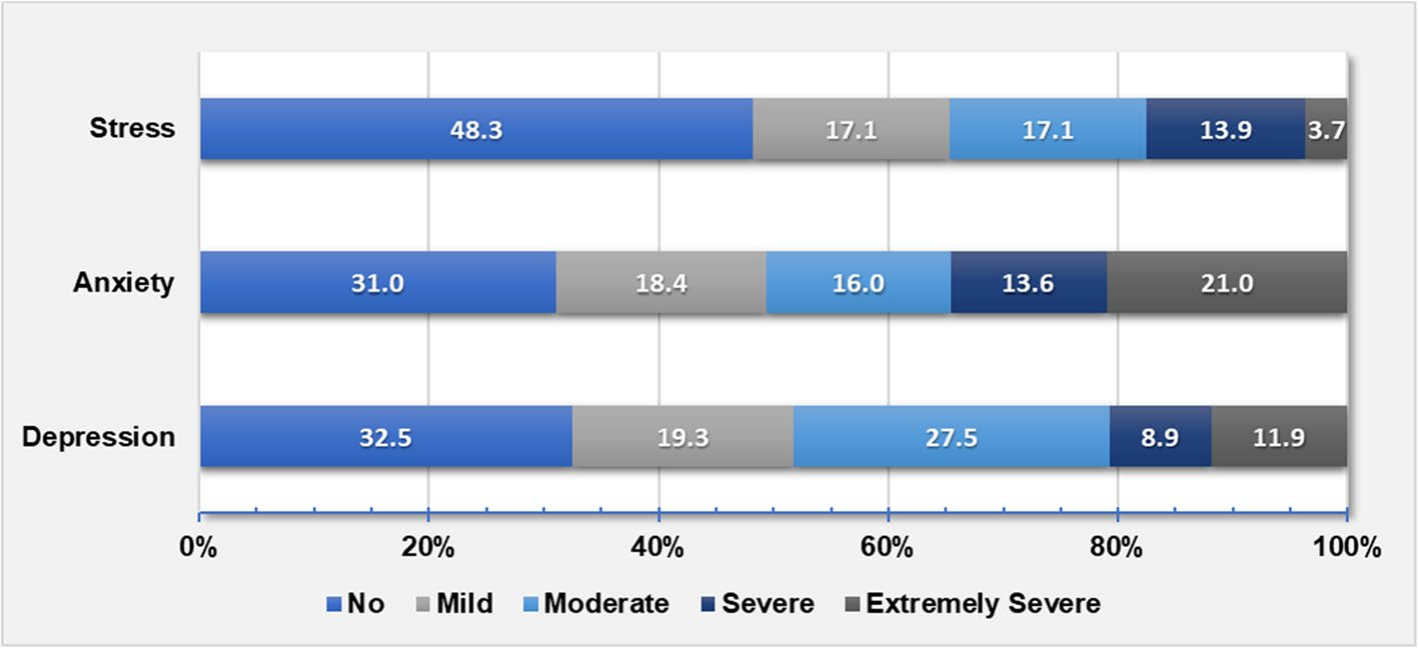
Proportions of the severity levels of depression, anxiety, and stress (*N* = 462)

**Fig. 3:**
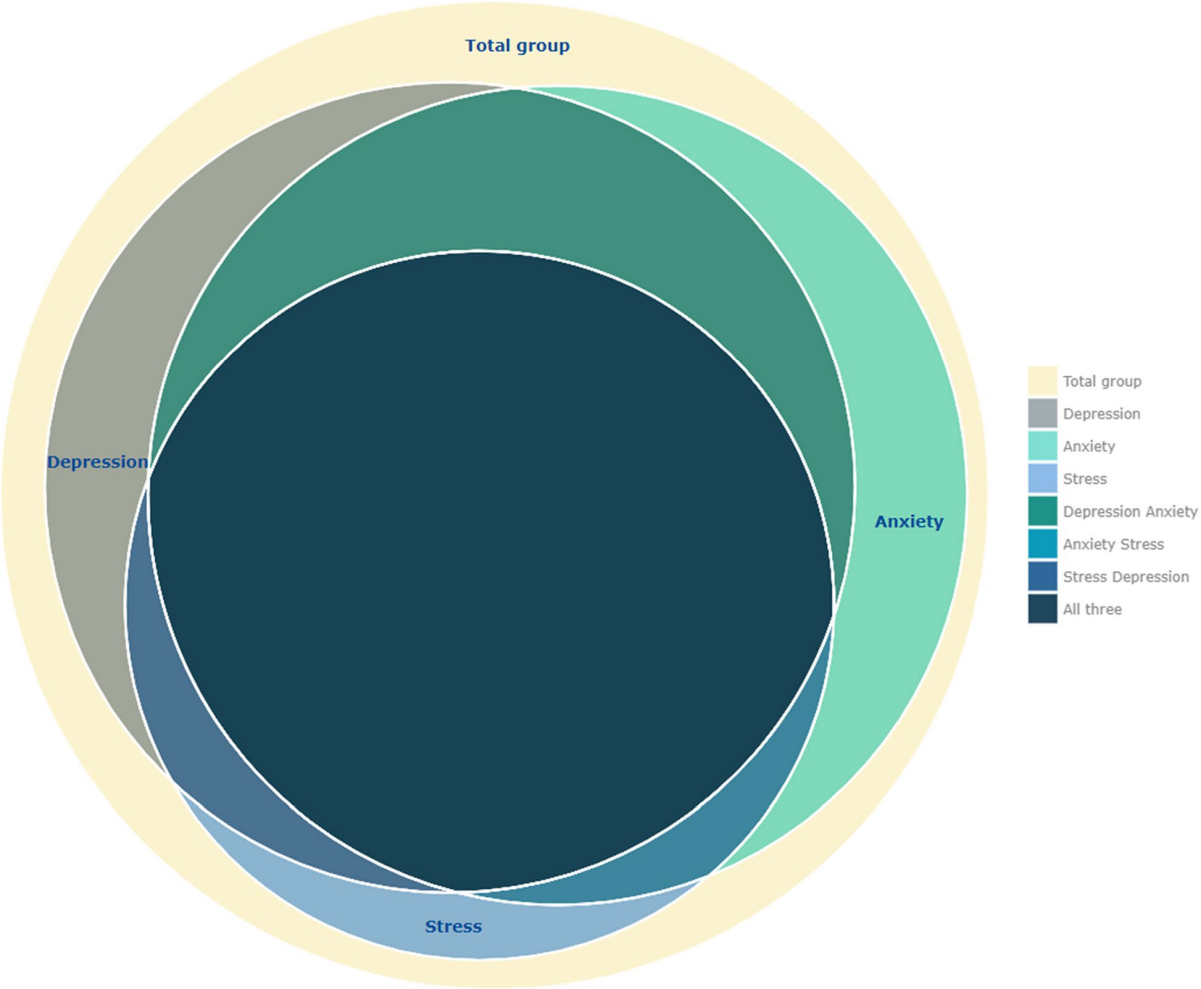
Concomitant presence of depression, anxiety, and/or stress. Depression and anxiety: 56.9%. Depression and stress: 46.8%. Anxiety and stress: 46.1%. The three states: 42.9%. None: 18.6%. An interactive copy of this figure can be found through this link: DAS_plot.html

Prevalence of suffering any level of depression, anxiety, and stress were 67.5% (312/462), 69.0% (319/462), and 51.7% (239/462); and of severe/extremely severe levels were 20.8, 34.6, and 17.6% respectively. Concomitant presence of the three states was reported by 42.9% (198/462) of staff (Table 1; Figs. 2 and 3).

#### Associated factors

We presented the bivariate analysis for the association of each of DAS with demographics, perceived job stress levels, and effort-reward imbalance in (Supplementary Table 3, Additional file 1), and with each individual WSS items in (Supplementary Table 4, Additional file 1). Effort-reward imbalance was significantly associated with depression (OR:2.76; 95%CI:1.85–4.12), anxiety (OR:2.40; 95%CI:1.61–3.60), and stress (OR:2.94; 95%CI:2.01–4.30). On multivariable analysis (Table 2), no differences were shown in the odds of depression, anxiety, or stress between the different job categories. The odds of depression were lower with the yearly increase in age (OR:0.98; 95%CI:0.96–0.99) and much higher with being in debt (OR:5.82; 95%CI:2.35–14.43) and just meeting routine expenses (OR:2.43; 95%CI:1.04–5.69) than with being able to save/invest money. There were steadily higher odds of depression (~ 5 up to ~ 33 folds) with higher perceived job stress levels. WSS items that significantly associated with depression were considering the work condition as unpleasant/unsafe (OR:1.74; 95%CI:1.38–2.19), difficulty communicating opinions/feelings to superiors (OR:1.32; 95%CI:1.12–1.55), interference of work with life (OR:1.64; 95%CI 1.38–1.95), and the less utilization of skills/talents at work (OR:1.23; 95%CI:1.05–1.44).

**Table 2:** Independent associates of depression, anxiety, and stress: multivariable ordinal regression

	Depression		Anxiety		Stress	
	Adjusted OR (95% CI) ^**a**^	***P***-Value	Adjusted OR (95% CI) ^**a**^	***P***-Value	Adjusted OR (95% CI) ^**a**^	***P***-Value
**Model 1**						
**Age**	0.98 (0.96-0.99)	**0.007**	0.99 (0.97-1.001)	0.069	0.97 (0.95-0.99)	**< 0.001**
**Gender**						
Men	Ref.		Ref.		Ref.	
Women	1.27 (0.88-1.83)	0.208	1.95 (1.36-2.81)	**< 0.001**	1.01 (0.69-1.49)	0.931
**Job categories**						
Unskilled workers	1.13 (0.29-4.41)	0.86	1.06 (0.31-3.65)	0.923	2.95 (0.75-11.59)	0.121
Technicians	2.69 (0.91-7.97)	0.075	1.32 (0.51-3.44)	0.57	1.91 (0.64-5.66)	0.245
Administrative	2.37 (0.82-6.81)	0.110	1.13 (0.46-2.80)	0.793	1.69 (0.60-4.80)	0.323
Engineers/IT professionals	Ref.		Ref.		Ref.	
**Income**						
In debt	5.82 (2.35-14.43)	**< 0.001**	3.84 (1.66-8.91)	**0.002**	3.01 (1.20-7.55)	**0.019**
Just meet routine expenses	2.43 (1.04-5.69)	**0.041**	2.26 (1.03-4.97)	**0.043**	1.64 (0.69-3.91)	0.265
Meet routine and emergency expenses	1.51 (0.615-3.73)	0.367	1.62 (0.70-3.76)	0.257	1.22 (0.48-3.10)	0.681
Able to save/invest money	Ref.		Ref.		Ref.	
**Job Stress Level**						
Relatively Calm	Ref.		Ref.		Ref.	
Fairly Low	1.70 (0.87-3.32)	0.124	1.60 (0.86-2.98)	0.135	1.36 (0.62-2.98)	0.446
Moderate	4.72 (2.44-9.14)	**< 0.001**	2.95 (1.62-5.37)	**< 0.001**	4.54 (2.17-9.48)	**< 0.001**
Severe	13.69 (6.77-27.70)	**< 0.001**	6.63 (3.47-12.65)	**< 0.001**	11.96 (5.53-25.89)	**< 0.001**
Potentially Dangerous	32.77 (13.43-79.98)	**< 0.001**	10.24 (4.44-23.59)	**< 0.001**	20.83 (8.25-52.62)	**< 0.001**
**Job Stressors (Model 2)**						
Unpleasant/unsafe work conditions	1.74 (1.38-2.19)	**< 0.001**	1.56 (1.26-1.93)	**< 0.001**	1.53 (1.21-1.94)	**< 0.001**
Job negatively affects physical/emotional wellbeing	0.95 (0.79-1.15)	0.600	1.11 (0.93-1.33)	0.238	1.04 (0.85-1.27)	0.728
Having much work/unreasonable deadlines	1.04 (0.88-1.24)	0.625	1.05 (0.89-1.24)	0.537	1.13 (0.94-1.35)	0.197
Difficult to express opinions/feelings to superiors	1.32 (1.12-1.55)	**0.001**	1.12 (0.96-1.30)	0.152	1.33 (1.12-1.58)	**0.001**
Job pressures interfere with family/personal life	1.64 (1.38-1.95)	**< 0.001**	1.38 (1.17-1.63)	**< 0.001**	1.62 (1.34-1.96)	**< 0.001**
^b^Having adequate control/input over work duties	0.99 (0.84-1.17)	0.889	1.09 (0.93-1.28)	0.288	0.99 (0.83-1.18)	0.917
^b^Receive appropriate recognition/rewards	0.93 (0.81-1.07)	0.291	0.88 (0.77-1.01)	0.071	0.91 (0.78-1.05)	0.193
^b^Utilize skills/talents to full extent at work	1.23 (1.05-1.44)	**0.010**	0.98 (0.84-1.14)	0.779	1.04 (0.88-1.23)	0.619

*IT* Information Technology, *OR* odds ratio, *CI* confidence interval

^a^Calculated using multivariable ordinal regression

^b^Reverse scoring was used

Higher odds of anxiety were shown among women than men (OR:1.95; 95%CI:1.36–2.81); and with being in debt (OR:3.84; 95%CI:1.66–8.91) and just meeting routine expenses (OR:2.26; 95%CI:1.03–4.97) than with being able to save/invest money. The odds of anxiety steadily showed higher values (~ 3 up to ~ 10 folds) with higher perceived job stress levels. Considering the work condition as unpleasant/unsafe (OR:1.56; 95%CI:1.26–1.93) and interference of work with life (OR:1.38; 95%CI:1.17–1.63) were the WSS items associated with anxiety.

Lower odds of stress were detected with the yearly increase of age (OR:0.97; 95%CI:0.95–0.99). Conversely, those who were in debt showed higher odds of stress (OR:3.01; 95%CI:1.20–7.55) than those being able to save/invest money. Again, steadily higher odds of stress were observed (~ 5 up to ~ 21 folds) with higher perceived job stress levels. Specifically, stress was associated with considering the work condition as unpleasant/unsafe (OR:1.53; 95%CI:1.21–1.94), difficulty communicating opinions/feelings to superiors (OR:1.33; 95%CI:1.12–1.58), and interference of work with life (OR:1.62; 95%CI:1.34–1.96) items of WSS.

## Discussion

### Main findings

We reported one of the earliest studies in Egypt measuring the emotional state of NCHS and its association with perceived job stress level. Unexpectedly, a big sector of NCHS were suffering emotional distress symptoms despite not being involved with direct patient care. Severe and extremely severe levels of depression and stress were reported by one of five; and of anxiety, by one of three participants. Regardless of their jobs, a third of NCHS recognized their work stress as severe or potentially dangerous with evidence of a financial effort-reward imbalance in half of them. DAS were strongly associated with low income and high perceived job stress. Particularly, feelings of unpleasant/unsafe work conditions and the work-life conflict were associated with the three states; while difficult communication with superiors was associated with depression and stress; and underutilization of skills with depression only.

### Emotional distress

Generally, studies conducted outside Egypt reported lower rates of emotional distress symptoms than those conducted inside it. In an Iranian population-based study, DAS symptoms were conveyed by 23.7, 26.3 and 30.3% of employed individuals respectively [17] and in an Indian study, by 19.7, 33.1, and 10.6% of government employees respectively [18]. Additionally, the proportion of NCHS who reported symptoms of depression in a Nigerian study was 7.5% [15]. During the era of COVID-19 pandemic, the pooled prevalence of DAS symptoms was 20.6, 27.0, and 36.4% respectively for staff other than physicians and nurses [16]. Like our results, studies from Egypt, that included different job categories, reported evidently higher proportions for the three states. Among medical students, the prevalence of DAS states ranged from ~ 60% to ~ 65% [20, 21] while among schoolteachers, anxiety was 67.5% and depression was 23.2% [19]. Prevalence of DAS among healthcare workers during COVID-19 pandemic was in ranges of ~ 59.0–69%, ~ 42.6–58.9%, and ~ 37.2–55.9% respectively [22, 23]. Furthermore, a larger study including multiple job categories reported that severe/very severe levels of DAS among professions in sectors other than health were 23.3%, 23.9, 14.7% respectively [29]. Taken within the context of the Egyptian studies and being conducted before the pandemic, our results confirmed the huge magnitude of emotional distress in our society that was not limited to healthcare professionals or attributed to the stressful situation of pandemic, a problem that may require much attention.

#### Associated factors

The association of the psychosocial working conditions and common mental health problems has long been documented [11, 30]. Supporting this association, we found that DAS symptoms were progressively more prevalent with higher perceived job stress levels, however, no differences were found in the different job categories of NCHS. Seemingly, the diversity of the jobs performed by NCHS had no effect on their feeling of DAS symptoms and was also not related to their perceived job stress level. Specifically, the likelihood of DAS symptoms was higher when jobs interfered with family/personal lives and when work conditions were perceived as unpleasant/unsafe. Literature also reported the relationship of work-family conflict and low mental health score [31]. In line with the studies that acknowledged the lack of managerial communication and support as a cause of depression, anxiety, and work-related stress [1, 11, 30], our study showed higher levels of depression and stress with difficulty in communicating with superiors. Also, the less frequent utilization of skills and talents at work was associated with depression among our study participants. Addressing hazards related to work-life conflict, communication at work, and leadership style is particularly required. Strategies that could be adopted are allowing flexible working time and location, encouraging open communication, and training on leadership skills [30]. Enhancing managers’ leadership skills will also help them find talented individuals to maximally utilize their capabilities.

Working conditions were not the only determinant of the workers’ emotional states; as only a third of NCHS, regardless of their job category, rated their jobs as severe/potentially dangerous. A very strong effector was also the income and its sufficiency to workers’ needs and capabilities. The relationship between financial suffering and emotional distress symptoms reported in literature [7–10] was confirmed by our finding of increasingly larger odds of DAS states with more serious levels of income insufficiency. A socioeconomic paradox was seen, as most of the staff were highly educated and belonging to the middle or high social class and, at the same time, receiving insufficient income. Additionally, an effort-reward imbalance, particularly financial, was shown among half of them. The paradox and imbalance may be caused by the greatly impacted Egyptian economy in years 2017 to 2019 with its resulting rise of prices that drained most of the population [12]. Our results confirmed the association of effort-reward imbalance with DAS symptoms that was previously reported in literature [11]. Contextually, our findings added more to the relative contribution of the financial aspect to mental health, not only among our study group but may also be nationally.

### Implications

In addition to the help in seeking psychiatric consultations for those experienced severe/extremely severe levels of distress, this study results motivated us to raise the issue of social working environment to the policy and decision makers to take the needed corrective actions. Also, we arranged to include the screening for mental health problems within the activity of occupational health clinic that serves all hospital staff.

### Strength and limitations

To our knowledge, this work was among the earliest to study symptoms of emotional distress among NCHS. Our study filled the knowledge gap about an important, but usually overlocked, sector of healthcare personnel particularly before the era of COVID-19 pandemic (data collection in 2019) when the studies were scarce on this group of workers. Publishing of Egyptian studies in late 2020 including clinical staff, has enabled a post-hoc comparison of our results across both the study participants (non-clinical versus clinical staff) and the time frame of the study conduct (before versus during COVID-19 pandemic). Also, some of the psychosocial working conditions that were related to workers’ mental health have been highlighted.

This study results are still valid even with this not recently collected dataset. The two main DAS-associated factors, namely job stress and economic conditions, are still present or even intensified. Hospital working conditions are still having the same, or even higher, stress level given the circumstances of COVID-19 pandemic. Additionally, the forementioned hard economic status in Egypt is expected to continue due to the adverse global economic developments that were recently aggravated by the war in Ukraine [32] and is expected to impact similar resource-limited countries too.

This research has some limitations. First, the cross-sectional design barely allows for making a causal inference. Second, the study setting in ASUHs, one of the largest Egyptian university hospitals serving a huge number of patients. This environment with a relatively higher job stress may entail cautious generalization of our results in other hospital settings. Third, due to the unbalanced representation of the various job categories in the included sample, we run a post hoc power calculation (Additional file 1) based on the distribution of depression within job categories shown in Table 1. Apart from “Technicians” group for whom the sample showed 83% power, the power was inadequate (< 80%) for all other job categories. This high level of β error can limit our conclusion about the absence of between job differences in the odds of emotional distress symptoms; however, it will most probably not jeopardize the within-jobs estimated proportions of DAS symptoms. Fourth, this study aimed to give an overview of emotional distress symptoms and differentiate between depression, anxiety, and stress using DASS-21 scale. For more in-depth investigation of mental health, we suggest using other tools such as Penn State Worry Questionnaire that distinguish between generalized anxiety disorder and other anxiety disorders. Also, the Worry Domain Questionnaire can be used, which systematically investigate the domains in which worry is experienced, including the work domain.

## Conclusion

Non-clinical hospital staff were commonly affected by symptoms of DAS with high rates of severe/very severe levels. Younger individuals suffered depression and stress more than older and women suffered anxiety more than men. Perceived Job stress level was strongly associated with emotional distress with an obvious higher likelihood with the higher levels. Measures to decrease job stress that are particularly related to work-life conflict, open communication with superiors, and finding talented personnel for best exploitation of their skills are needed. Most of participants received insufficient income despite belonging to the middle and high social class; and an effort-reward imbalance, particularly financial, was shown. Insufficient income was a strong correlate with emotional distress, and mitigation measures are urgently needed.

## Supplementary Information

**Additional file 1: Supplementary Table 1.** Insufficient income correlates. **Supplementary Table 2.** Correlates of severe/potentially dangerous job stress level. **Supplementary Table 3.** Depression, Anxiety and Stress: Bivariate analysis by demographic characteristics and job stress levels. **Supplementary Table 4.** Depression, Anxiety and Stress: Bivariate analysis by individual job stressors. Power calculation. The power calculation for the sample to detect a difference between job categories. Calculation is based on the distribution of depression within job categories (snapshots from output of OpenEpi power calculator).

## Data Availability

The data underlying this article will be shared on reasonable request to the corresponding author.

## Abbreviations

DAS: Depression, Anxiety, and Stress
COVID-19: Corona virus disease 2019
NCHS: Non-clinical hospital staff
ASUHs: Ain-Shams University Hospitals
IT: Information technology
DASS-21: Depression Anxiety Stress Scale-21
DASS-42: Depression Anxiety Stress Scale-42
WSS: Workplace Stress Scale
IQR: Interquartile range
OR: Odds ratio
CI: Confidence interval
