# Supplementary material for: Emotional distress symptoms and their determinants: screening of non-clinical hospital staff in an Egyptian University hospital
Source: BMC Psychiatry. 2022 Dec 15;22:793. doi: 10.1186/s12888-022-04463-4 (PMC9754778; doi:10.1186/s12888-022-04463-4)
Supplement: Supplementary file 1 — Additional file 1: Supplementary Table 1. Insufficient income correlates. Supplementary Table 2. Correlates of severe/potentially dangerous job stress level. Supplementary Table 3. Depression, Anxiety and Stress: Bivariate analysis by demographic characteristics and job stress levels. Supplementary Table 4. Depression, Anxiety and Stress: Bivariate analysis by individual job stressors. Power calculation. The power calculation for the sample to detect a difference between job categories. Calculation is based on the distribution of depression within job categories (snapshots from output of OpenEpi power calculator). [file 12888_2022_4463_MOESM1_ESM.pdf]

Emotional distress symptoms and their determinants: Screening of non-clinical hospital staff in an Egyptian University Hospital

Noha M. Ibrahim,[1] M.D., Dina A. Gamal-Elden, [1] M.D., Mohsen A. Gadallah, [1] M.D., Sahar K. Kandil,[1] M.D.*

[1] Department of Community, Environmental, and Occupational Medicine, Faculty of Medicine, Ain Shams University, Cairo, Egypt

*** Corresponding Author**

Name: Sahar K. Kandil, M.D.

ORCID: https://orcid.org/0000-0001-9433-659X

E-mail: saharkandil@med.asu.edu.eg

Address: Faculty of Medicine, Ain Shams University, Cairo, Egypt, 38 Ramses St., Abbassia square, Post code 11566.

Tel/Fax: 00202 24346888

Supplementary Tables

Supplementary table 1: Insufficient income correlates

	Total	Insufficient income			
	**(N=462) (column%)**	**N=335 (72.5%) (column%)**	**OR (95% CI) ***	**P-Value**	**(row %)**
**Age groups**					
18 – 30 years	70 (15.2)	47 (14.0)	Ref.		67.1
31 – 45 years	164 (35.5)	123 (36.7)	1.11 (0.57-2.17)	0.763	75.0
46 – 60 years	228 (49.4)	165 (49.3)	0.87 (0.46-1.66)	0.675	72.4
**Gender**					
Men	171 (37.0)	124 (37.0)	Ref.		72.5
Women	291 (63.0)	211 (63.0)	0.94 (0.60-1.47)	0.770	72.5
**Level of education**					
Less than university	183 (39.6)	144 (43.0)	Ref.		78.7
University or higher	279 (60.4)	191 (57.0)	0.52 (0.32-0.84)	**0.007**	68.5
**Job categories**					
Workers	17 (3.7)	13 (3.9)	1.99 (0.44-9.05)	0.372	76.5
Technicians	81 (17.5)	55 (16.4)	1.63 (0.57-4.67)	0.367	67.9
Administrative	344 (74.5)	257 (76.7)	2.89 (1.06-7.87)	**0.037**	74.7
Engineers and IT professionals	20 (4.3)	10 (3.0)	Ref.		50.0

* Multivariable logistic regression was used including age groups, gender, educational level, and job categories in the model. IT: Information Technology. OR: odds ratio. CI: confidence interval

Supplementary Table 2: Correlates of severe/potentially dangerous job stress level

	Total	Severe/Potentially dangerous job stress			
	N. = 462	N. = 139 (column%)	OR (95% CI)*	P-Value	Row %
**Age groups**					
18 – 30 years	70 (15.2)	24 (17.3)	Ref.		34.3
31 – 45 years	164 (35.5)	53 (38.1)	0.92 (0.51-1.66)	0.769	32.3
46 – 60 years	228 (49.4)	62 (44.6)	0.72 (0.40-1.27)	0.253	27.2
**Gender**					
Women	291 (63.0)	86 (61.9)	Ref.		29.6
Men	171 (37.0)	53 (38.1)	1.07 (0.71-1.61)	0.744	31.0
**Job categories**					
Workers	17 (3.7)	4 (2.9)	1.74 (0.33-9.19)	0.512	23.5
Technicians	81 (17.5)	28 (20.1)	2.99 (0.81-11.10)	0.101	34.6
Administrative	344 (74.5)	104 (74.8)	2.46 (0.70-8.56)	0.159	30.2
Engineers and IT professionals	20 (4.3)	3 (2.2)	Ref.		15.0

*Binary logistic regression was used

Supplementary Table 3: Depression, Anxiety and Stress: Bivariate analysis by demographic characteristics and job stress levels

	Total group	Depression (Any level)			Anxiety (Any level)			Stress (Any level)		
	**N. = 462**	N.= **312** Overall prevalence = **67.5**%			N.= **319** Overall prevalence = **69.0%**			N.= **239** Overall prevalence = **51.7%**		
		95% CI (63.2% - 71.7%)			95% CI (64.7% - 73.1%)			95% CI (47.1% - 56.3%)		
	n. (column %)	n. (column %)	Unadjusted OR (95% CI) *	P-Value	n. (column %)	Unadjusted OR (95% CI) *	P-Value	n. (column %)	Unadjusted OR (95% CI) *	P-Value
**Age**										
18 – 30 years	70 (15.2)	51 (16.3)	Ref.		52 (16.3)	Ref.		46 (19.2)	Ref.	
31 – 45 years	164 (35.5)	115 (36.9)	0.87 (047-1.63)	0.673	114 (35.7)	0.79 (0.42-1.48)	0.462	95 (39.7)	0.72 (0.40-1.29)	0.266
46 – 60 years	228 (49.4)	146 (46.8)	0.66 (0.37-1.20)	0.174	153 (48.0)	0.71 (0.39-1.29)	0.258	98 (41.0)	0.39 (0.23-0.69)	**0.001**
**Gender**										
Men	171 (37.0)	110 (35.3)	Ref.		104 (32.6)	Ref.		91 (38.1)	Ref.	
Women	291 (63.0)	202 (64.7)	1.26 (0.84-1.88)	0.260	215 (67.4)	1.82 (1.22-2.73)	**0.004**	148 (61.9)	0.91 (0.62-1.33)	0.624
**Marital status**										
Un-married	89 (19.3)	62 (19.9)	Ref.		58 (18.2)	Ref.		46 (19.2)	Ref.	
Married	373 (80.7)	250 (80.1)	0.89 (0.54-1.46)	0.633	261 (81.8)	1.25 (0.76-2.03)	0.379	193 (80.8)	1.00 (0.63-1.59)	0.992
**Residence**										
Rural/urban slum	60 (13.0)	36 (11.5)	Ref.		46 (14.4)	Ref.		38 (15.9)	Ref.	
Urban	402 (87.0)	276 (88.5)	1.46 (0.84-2.55)		273 (85.6)	0.64 (0.34-1.21)	0.174	201 (84.1)	0.58 (0.33-1.01)	0.056
**Educational level**										
Less than university	183 (39.6)	125 (40.1)	Ref.		128 (40.1)	Ref.		95 (39.7)	Ref.	
University or higher	279 (60.4)	187 (59.9)	0.94 (0.63-1.41)	0.774	191 (59.9)	0.93 (0.62-1.40)	0.735	144 (60.3)	0.99 (0.68-1.44)	0.95
**Job categories**										
Workers	17 (3.7)	12 (3.8)	3.60 (0.91-14.23)	0.068	12 (3.8)	1.96 (0.50-7.69)	0.333	12 (5.0)	4.46 (1.11-17.90)	**0.035**
Technicians	81 (17.5)	60 (19.2)	4.29 (1.54-11.93)	**0.005**	59 (18.5)	2.19 (0.80-6.01)	0.126	49 (20.5)	2.84 (1.02-7.90)	**0.045**
Administrative	344 (74.5)	232 (74.4)	3.11 (1.24-7.82)	**0.016**	237 (74.3)	1.81 (0.73-4.50)	0.2	171 (71.5)	1.84 (0.72-4.71)	0.207
Engineers and IT professionals	20 (4.3)	8 (2.6)	Ref.		11 (3.4)	Ref.		7 (2.9)	Ref.	

**Income**										
In debt	97 (21.0)	82 (26.3)	7.95 (3.09-20.45)	**<0.0001**	78 (24.5)	5.13 (2.07-12.75)	**<0.0001**	65 (27.2)	3.45 (1.42-8.40)	**0.006**
Just meet routine expenses	238 (51.5)	167 (53.5)	3.42 (1.51-7.74)	**0.003**	169 (53.0)	3.06 (1.36-6.88)	**0.007**	125 (52.3)	1.88 (0.83-4.28)	0.132
Meet routine and emergency expenses	100 (21.6)	52 (16.7)	1.58 (0.67-3.73)	0.301	60 (18.8)	1.88 (0.80-4.42)	0.151	39 (16.3)	1.09 (0.45-2.62)	0.853
Able to save/invest money	27 (5.8)	11 (3.5)	Ref.		12 (3.8)	Ref.		10 (4.2)	Ref.	
**Socioeconomic status**										
Low	18 (3.9)	12 (3.8)	1.06 (0.36-3.14)	0.918	14 (4.4)	2.19 (0.66-7.27)	0.202	14 (5.9)	4.30 (1.30-14.24)	**0.017**
Middle	366 (79.2)	249 (79.8)	1.13 (0.67-1.89)	0.650	257 (80.6)	1.47 (0.89-2.45)	0.135	190 (79.5)	1.33 (0.81-2.17)	0.260
High	78 (16.9)	51 (16.3)	Ref.		48 (15.0)	Ref.		35 (14.6)	Ref.	
**Job Stress Level**										
Relatively Calm	57 (12.3)	19 (6.1)	Ref.		25 (7.8)	Ref.		12 (5.0)	Ref.	
Fairly Low	117 (25.3)	60 (19.2)	2.11 (1.09-4.07)	**0.027**	67 (21.0)	1.72 (0.91-3.25)	0.098	31 (13.0)	1.35 (0.63-2.88)	0.436
Moderate	149 (32.3)	105 (33.7)	4.77 (2.48-9.18)	**<0.0001**	107 (33.5)	3.26 (1.73-6.14)	**<0.0001**	84 (35.1)	4.85 (2.37-9.90)	**<0.0001**
Severe	104 (22.5)	95 (30.4)	21.11 (8.78-50.79)	**<0.0001**	88 (27.6)	7.04 (3.34-14.85)	**<0.0001**	82 (34.3)	13.98 (6.33-30.85)	**<0.0001**
Potentially Dangerous	35 (7.6)	33 (10.6)	33.00 (7.15-152.38)	**<0.0001**	32 (10.0)	13.65 (3.74-49.80)	**<0.0001**	30 (12.6)	22.5 (7.19-70.42)	**<0.0001**
**Effort-reward imbalance**										
Yes (any)	253 (54.8)	196 (62.8)	2.76 (1.85-4.12)	**<0.0001**	196 (61.4)	2.40 (1.61-3.60)	**<0.0001**	161 (67.4)	2.94 (2.01-4.30)	**<0.0001**
Financial	229 (49.6)	179 (57.4)	2.69 (1.79-4.04)	**<0.0001**	179 (56.1)	2.38 (1.58-3.58)	**<0.0001**	145 (60.7)	2.55 (1.75-3.71)	**<0.0001**
Emotional	146 (31.6)	114 (36.5)	2.12 (1.35-3.34)	**0.001**	118 (37.0)	2.41 (1.51-3.86)	**<0.0001**	102 (42.7)	3.03 (1.99-4.60)	**<0.0001**

* Binary logistic regression was used for their calculation

Supplementary Table 4: Depression, Anxiety and Stress: Bivariate analysis by individual job stressors

	Total group	Depression (Any level)			Anxiety (Any level)			Stress (Any level)		
	**N. = 462**	N.= **312** Overall prevalence = **67.5%**			N.= **319** Overall prevalence = **69.0%**			N.= **239** Overall prevalence = **51.7%**		
		95% CI (63.2% - 71.7%)			95% CI (64.7% - 73.1%)			95% CI (47.1% - 56.3%)		
	n. (column %)	n. (column %)	Unadjusted OR (95% CI) *	P-Value	n. (column %)	Unadjusted OR (95% CI) *	P-Value	n. (column %)	Unadjusted OR (95% CI) *	P-Value
**Unpleasant/unsafe work conditions**										
Never	47 (10.2)	16 (5.1)	Ref.		19 (6.0)	Ref.		10 (4.2)	Ref.	
Rarely	69 (14.9)	31 (9.9)	1.58 (0.73-3.41)	0.242	38 (11.9)	1.81 (0.85-3.83)	0.123	21 (8.8)	1.62 (0.68-3.85)	0.276
Sometimes	221 (47.8)	153 (49.0)	4.36 (2.24-8.50)	<0.0001	152 (47.6)	3.25 (1.70-6.21)	<0.0001	107 (44.8)	3.47 (1.65-7.33)	0.001
Often	71 (15.4)	61 (19.6)	11.82 (4.80-29.09)	<0.0001	61 (19.1)	8.99 (3.70-21.82)	<0.0001	60 (25.1)	20.18 (7.81-52.15)	<0.0001
Very often	54 (11.7)	51 (16.3)	32.94 (8.88-122.25)	<0.0001	49 (15.4)	14.44 (4.86-42.92)	<0.0001	41 (17.2)	11.67 (4.57-29.77)	<0.0001
**Job negatively affects physical/emotional wellbeing**										
Never	101 (21.9)	49 (15.7)	Ref.		50 (15.7)	Ref.		28 (11.7)	Ref.	
Rarely	60 (13.0)	35 (11.2)	1.49 (0.78-2.83)	0.299	39 (12.2)	1.89 (0.98-3.66)	0.057	24 (10.0)	1.74 (0.88-3.42)	0.109
Sometimes	163 (35.3)	105 (33.7)	1.92 (1.16-3.18)	0.011	109 (34.2)	2.06 (1.24-3.42)	0.005	78 (32.6)	2.39 (1.40-4.08)	0.001
Often	72 (15.6)	62 (19.9)	6.58 (3.04-14.26)	<0.0001	60 (18.8)	5.10 (2.45-10.61)	<0.0001	59 (24.7)	11.83 (5.64-24.85)	<0.0001
Very often	66 (14.3)	61 (19.6)	12.95 (4.80-34.90)	<0.0001	61 (19.1)	12.44 (4.62-33.55)	<0.0001	50 (20.9)	8.15 (4.00-16.60)	<0.0001
**Having much work/unreasonable deadlines**										
Never	71 (15.4)	31 (9.9)	Ref.		35 (11.0)	Ref.		17 (7.1)	Ref.	
Rarely	90 (19.5)	60 (19.2)	2.58 (1.36-4.90)	0.004	59 (18.5)	1.96 (1.04-3.70)	0.039	42 (17.6)	2.78 (1.40-5.51)	0.003
Sometimes	168 (36.4)	118 (37.8)	3.05 (1.72-5.41)	<0.0001	123 (38.6)	2.81 (1.58-5.01)	<0.0001	92 (38.5)	3.85 (2.06-7.18)	<0.0001
Often	73 (15.8)	52 (16.7)	3.20 (1.60-6.37)	0.001	54 (16.9)	2.92 (1.45-5.89)	0.003	42 (17.6)	4.30 (2.10-8.81)	<0.0001
Very often	60 (13.0)	51 (16.3)	7.31 (3.13-17.10)	<0.0001	48 (15.0)	4.11 (1.88-9.02)	<0.0001	46 (19.2)	10.44 (4.65-23.45)	<0.0001
**Difficult to express opinions/feelings about job conditions to superiors**										
Never	103 (22.3)	41 (13.1)	Ref.		57 (17.9)	Ref.		25 (10.5)	Ref.	
Rarely	70 (15.2)	40 (12.8)	2.02 (1.09-3.73)	0.026	43 (13.5)	1.29 (0.69-2.39)	0.426	29 (12.1)	2.21 (1.15-4.25)	0.018
Sometimes	113 (24.5)	84 (26.9)	4.38 (2.46-7.81)	<0.0001	79 (24.8)	1.88 (1.07-3.28)	0.028	56 (23.4)	3.07 (1.71-5.49)	<0.0001
Often	98 (21.2)	73 (23.4)	4.42 (2.42-8.06)	<0.0001	72 (22.6)	2.24 (1.24-4.05)	0.008	64 (26.8)	5.87 (3.18-10.84)	<0.0001
Very often	78 (16.9)	74 (23.7)	27.98 (9.49-82.44)	<0.0001	68 (21.3)	5.49 (2.54-11.84)	<0.0001	65 (27.2)	15.60 (7.39-32.92)	<0.0001

**Job pressures interfere with family/personal life**									
Never	105 (22.7)	43 (13.8)	Ref.		47 (14.7)	Ref.		17 (7.1)	Ref.	
Rarely	81 (17.5)	43 (13.8)	1.63 (0.91-2.93)	0.101	57 (17.9)	2.93 (1.59-5.41)	0.001	30 (12.6)	3.05 (1.53-6.06)	0.002
Sometimes	135 (29.2)	104 (33.3)	4.84 (2.77-8.46)	<0.0001	95 (29.8)	2.93 (1.72-5.00)	<0.0001	86 (36.0)	9.09 (4.86-17.00)	<0.0001
Often	75 (16.2)	61 (19.6)	6.28 (3.12-12.64)	<0.0001	61 (19.1)	5.38 (2.68-10.79)	<0.0001	51 (21.3)	11.00 (5.41-22.39)	<0.0001
Very often	66 (14.3)	61 (19.6)	17.59 (6.53-47.40)	<0.0001	59 (18.5)	10.40 (4.35-24.90)	<0.0001	55 (23.0)	25.88 (11.29-59.35)	<0.0001
**Having adequate control/input over work duties** †									
Never	32 (6.9)	16 (5.1)	0.74 (0.35-1.58)	0.439	18 (5.6)	0.70 (0.33-1.50)	0.359	10 (4.2)	0.52 (0.23-1.16)	0.112
Rarely	34 (7.4)	25 (8.0)	2.06 (0.91-4.68)	0.083	27 (8.5)	2.10 (0.86-5.09)	0.102	23 (9.6)	2.40 (1.10-5.21)	0.027
Sometimes	66 (14.3)	51 (16.3)	2.53 (1.32-4.83)	0.005	51 (16.0)	1.85 (0.96-3.56)	0.065	32 (13.4)	1.08 (0.61-1.90)	0.793
Often	154 (33.3)	119 (38.1)	2.53 (1.56-4.08)	<0.0001	109 (34.2)	1.32 (0.83-2.10)	0.245	92 (38.5)	1.70 (1.10-2.64)	0.017
Very often	176 (38.1)	101 (32.4)	Ref.		114 (35.7)	Ref.		82 (34.3)	Ref.	
**Receive appropriate recognition/rewards for good performance** †									
Never	118 (25.5)	91 (29.2)	2.05 (1.09-3.88)	0.027	90 (28.2)	1.54 (0.81-2.94)	0.188	73 (30.5)	2.38 (1.31-4.31)	0.004
Rarely	100 (21.6)	69 (22.1)	1.36 (0.72-2.55)	0.347	73 (22.9)	1.30 (0.67-2.50)	0.437	61 (25.5)	2.29 (1.24-4.24)	0.008
Sometimes	106 (22.9)	69 (22.1)	1.14 (0.61-2.10)	0.687	66 (20.7)	0.79 (0.42-1.48)	0.465	48 (20.1)	1.21 (0.67-2.22)	0.528
Often	64 (13.9)	37 (11.9)	0.83 (0.42-1.65)	0.603	40 (12.5)	0.80 (0.40-1.61)	0.533	27 (11.3)	1.07 (0.54-2.11)	0.845
Very often	74 (16.0)	46 (14.7)	Ref.		50 (15.7)	Ref.		30 (12.6)	Ref.	
**Utilize skills/talents to full extent at work** †									
Never	41 (8.9)	32 (10.3)	2.96 (1.33-6.63)	0.008	30 (9.4)	1.79 (0.83-3.84)	0.135	22 (9.2)	1.59 (0.79-3.17)	0.192
Rarely	38 (8.2)	31 (9.9)	3.69 (1.53-8.89)	0.004	24 (7.5)	1.12 (0.54-2.34)	0.754	24 (10.0)	2.35 (1.13-4.88)	0.022
Sometimes	104 (22.5)	86 (27.6)	3.98 (2.19-7.25)	<0.0001	87 (27.3)	3.36 (1.82-6.19)	<0.0001	59 (24.7)	1.80 (1.09-2.97)	0.023
Often	125 (27.1)	79 (25.3)	1.43 (0.88-2.32)	0.145	85 (26.6)	1.39 (0.85-2.29)	0.189	69 (28.9)	1.69 (1.05-2.72)	0.031
Very often	154 (33.3)	84 (26.9)	Ref.		93 (29.2)	Ref.		65 (27.2)	Ref.	

* Binary logistic regression was used for their calculation
† Reverse scoring was used

Power calculation

The power calculation for the sample to detect a difference between job categories. Calculation is based on the distribution of depression within job categories (snapshots from output of OpenEpi power calculator).

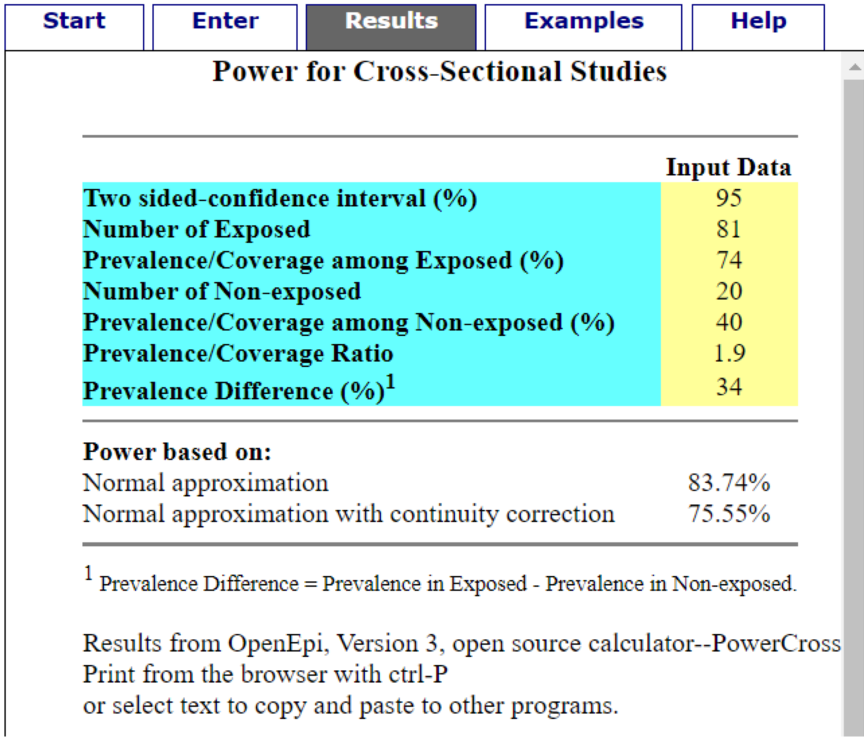

Start | Enter | Results | Examples | Help

Power for Cross-Sectional Studies

	Input Data
Two sided-confidence interval (%)	95
Number of Exposed	81
Prevalence/Coverage among Exposed (%)	74
Number of Non-exposed	20
Prevalence/Coverage among Non-exposed (%)	40
Prevalence/Coverage Ratio	1.9
Prevalence Difference (%)[1]	34

**Power based on:**	
Normal approximation	83.74%
Normal approximation with continuity correction	75.55%

[1] Prevalence Difference = Prevalence in Exposed - Prevalence in Non-exposed.

Results from OpenEpi, Version 3, open source calculator--PowerCross
Print from the browser with ctrl-P
or select text to copy and paste to other programs.

Technicians vs. Engineers/IT professional

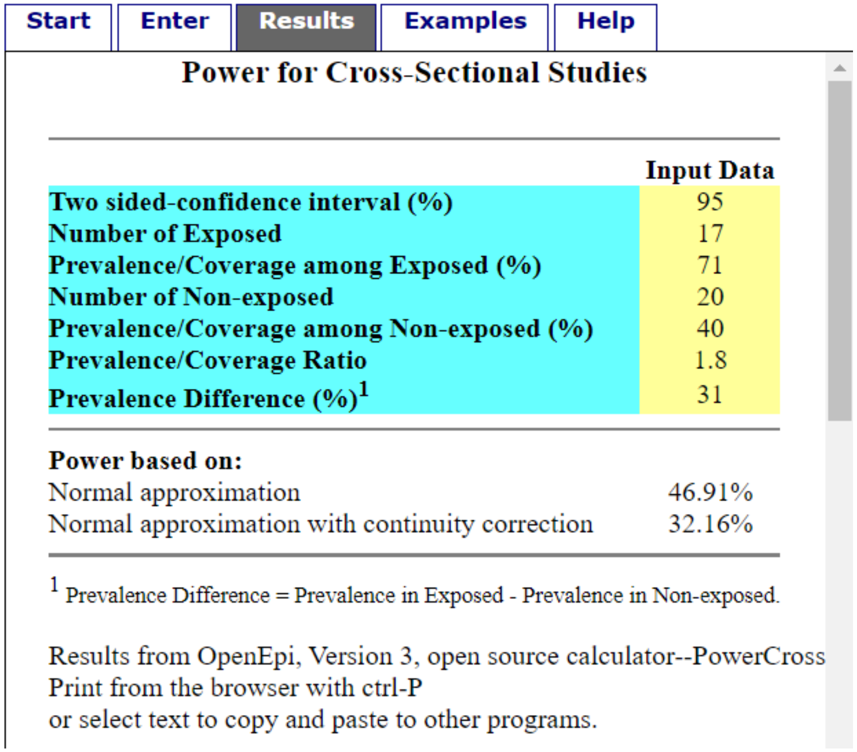

Start | Enter | Results | Examples | Help

Power for Cross-Sectional Studies

	Input Data
Two sided-confidence interval (%)	95
Number of Exposed	17
Prevalence/Coverage among Exposed (%)	71
Number of Non-exposed	20
Prevalence/Coverage among Non-exposed (%)	40
Prevalence/Coverage Ratio	1.8
Prevalence Difference (%)[1]	31

**Power based on:**	
Normal approximation	46.91%
Normal approximation with continuity correction	32.16%

[1] Prevalence Difference = Prevalence in Exposed - Prevalence in Non-exposed.

Results from OpenEpi, Version 3, open source calculator--PowerCross
Print from the browser with ctrl-P
or select text to copy and paste to other programs.

Unskilled workers vs. Engineers/IT professional

Start | Enter | Results | Examples | Help

Power for Cross-Sectional Studies

	Input Data
**Two sided-confidence interval (%)**	95
**Number of Exposed**	344
**Prevalence/Coverage among Exposed (%)**	67
**Number of Non-exposed**	20
**Prevalence/Coverage among Non-exposed (%)**	40
**Prevalence/Coverage Ratio**	1.7
**Prevalence Difference (%)**[1]	27

**Power based on:**	
Normal approximation	69.64%
Normal approximation with continuity correction	60.13%

[1] Prevalence Difference = Prevalence in Exposed - Prevalence in Non-exposed.

Results from OpenEpi, Version 3, open source calculator--PowerCross
Print from the browser with ctrl-P
or select text to copy and paste to other programs.

Administrative vs. Engineers/IT professional

Start | Enter | Results | Examples | Help

Power for Cross-Sectional Studies

	Input Data
**Two sided-confidence interval (%)**	95
**Number of Exposed**	20
**Prevalence/Coverage among Exposed (%)**	40
**Number of Non-exposed**	442
**Prevalence/Coverage among Non-exposed (%)**	69
**Prevalence/Coverage Ratio**	0.58
**Prevalence Difference (%)**[1]	-29

**Power based on:**	
Normal approximation	77.64%
Normal approximation with continuity correction	69.19%

[1] Prevalence Difference = Prevalence in Exposed - Prevalence in Non-exposed.

Results from OpenEpi, Version 3, open source calculator--PowerCross
Print from the browser with ctrl-P
or select text to copy and paste to other programs.

Engineers/IT professional vs. Other job categories
